# Nutrition-Based Counseling and Interventions for Chronic Disease Prevention

**DOI:** 10.3390/nu18213529

**Published:** 2026-10-27

**Authors:** Evridiki Patelarou, Konstantinos Giakoumidakis

**Affiliations:** 1 Department of Nursing, School of Health Sciences, Hellenic Mediterranean University, 71410 Heraklion, Greece; epatelarou@hmu.gr; 2 Laboratory of Evidence-Based Healthcare, Education and Clinical Protocols, Department of Nursing, School of Health Sciences, Hellenic Mediterranean University, 71410 Heraklion, Greece

## 1. Introduction

Chronic diseases, such as cardiovascular disease, type 2 diabetes mellitus, obesity, and chronic kidney disease, are the leading causes of morbidity and mortality worldwide. They constitute a significant burden on healthcare systems and on the quality of life of affected individuals [1], a large part of which is associated with modifiable risk factors, with diet playing a central role [1]. Incorporating nutritional counseling into routine patient care is a key strategy for primary and secondary prevention, particularly among obese individuals and those at increased cardiovascular risk [2]. Numerous studies have confirmed that interventions aimed at improving dietary behavior among patients living with chronic diseases, or at risk of developing them, are associated with better outcomes, although their effectiveness depends largely on their design, duration, and degree of personalization [3].

Within the interaction between healthcare professionals and patients, nutritional counseling is not limited to the dissemination of knowledge; it aims to empower patients to manage their chronic condition by adopting lasting lifestyle changes [4,5]. There is evidence that numerous psychological, behavioral, and social factors influence eating behavior and contribute to the effectiveness of nutritional interventions [6]. Moreover, nutrition plays a pivotal role in healthy aging with adherence to beneficial habits; for instance, the Mediterranean Diet is strongly associated with improved functionality and quality of life among older adults [7]. This finding has significant value in the era of an increasingly ageing world population.

The Special Issue of *Nutrients*, entitled “Nutrition-Based Counseling and Interventions for Chronic Disease Prevention”, aims to provide healthcare professionals with practical, up-to-date, and evidence-based insights into effective strategies for the optimal management of chronic diseases through sustainable dietary changes, enhancing quality of life and reducing disease burden in the long term. The eight papers included cover a wide range of clinical conditions, patient populations and disciplines, highlighting the need for a holistic, collaborative, and personalized approach.

## 2. Key Contributions

Chatziefstratiou et al. (Contribution 1) investigated the relationship between dietary habits and self-care behavior in 137 outpatients with heart failure in Greece. Although overall dietary quality was not significantly associated with self-care after adjustment for demographic factors, the saturated fatty acid score remained independently associated, suggesting that specific aspects of dietary behavior may warrant targeted counseling in this population.

Focusing on metabolic syndrome, Kim and Kwak (Contribution 2) analyzed data from nearly 15,000 Korean adults and found that high carbohydrate intake (>65% of energy intake) and low fat intake (<15%) were associated with a higher likelihood of metabolic syndrome, whereas the theoretical isocaloric replacement of carbohydrate with fat or protein was associated with reduced risk, with significant interactions by sex and age.

Ghouili et al. (Contribution 3) developed a machine learning framework to classify the nutritional status of 4232 adolescents in Tunisia, taking biological maturation into account. The cost-sensitive random forest model achieved high classification accuracy, with body mass emerging as the most important predictor—an approach offering direct application in school-based preventive screening programs.

The study by López-Fernández-Roldán et al. (Contribution 4) examined the prevalence of gastrointestinal symptoms in more than 34,000 adults. These symptoms were associated with factors such as age, sex, depression, and the intake of protein and sugary products. The authors concluded that higher intake of dietary fiber and legumes is beneficial, leading to lower risk, and also underlined the importance of a holistic, physical, and psychological approach.

Croce et al. (Contribution 5) examined body composition and dietary habits in patients with newly diagnosed Graves’ disease compared with euthyroid controls. Patients showed elevated extracellular water and reduced body cell mass, while the standardized phase angle showed an inverse relationship with disease severity, supporting its clinical use as a tool for evaluating nutritional status in thyrotoxicosis. Notably, pre-morbid weight changes did not correlate with disease severity and may be explained by increased caloric intake.

Ben Othman et al. (Contribution 6) investigated fat taste sensitivity among 100 Tunisian children. They found that obese children had significantly higher fat detection thresholds than non-obese children, suggesting a possible link between the biological mechanisms of taste perception, dietary preferences, and obesity risk.

Bouloukaki et al. (Contribution 7) examined a large cohort of patients with obstructive sleep apnea and identified an association between vitamin B12 status and worse objective sleep quality, with possible implications for cardiovascular disease; further research is needed to confirm a causal relationship and to explore the mechanism linking vitamin B12 to sleep parameters and cardiovascular outcomes.

Finally, the review by Tai et al. (Contribution 8) on sarcopenia in patients with hepatocellular carcinoma synthesized current knowledge on the biological mechanisms of muscle mass loss, translating the literature into practical principles of supportive care—adequate energy and protein intake, optimized meal distribution, and targeted supplementation—while also emphasizing the need for individualization according to disease stage and liver function.

## 3. Conclusions

Despite the heterogeneity of the eight papers in this collection in terms of target population, methodology, and clinical context, a common conclusion emerges: the effective prevention of chronic diseases through nutrition relies on personalized, evidence-based interventions that take into account biological, psychological, developmental, and social factors, rather than on generic recommendations [8]. Integrating digital tools into nutritional counseling is a particularly promising direction, as it enables continuous monitoring and the adaptation of interventions to individual patients [9,10]. At the same time, emphasis on dietary patterns with evident benefits for healthy aging, such as the Mediterranean Diet, remains the main pillar for the prevention of age-related diseases [7,11].

Future research should further investigate the mechanisms of diet-disease interaction and evaluate nutritional care models across settings, from clinical practice to public health and schools [12]. Strengthening interdisciplinary collaboration among physicians, dietitians, nurses, and psychologists is essential for the successful implementation of these care models in everyday clinical practice [13,14]. Establishing nutrition as a pillar of evidence-based clinical practice can contribute to more effective management of chronic disease and improved quality of life for patients and their families.
